# On Interpretational Questions for Quantum-Like Modeling of Social Lasing

**DOI:** 10.3390/e20120921

**Published:** 2018-12-02

**Authors:** Andrei Khrennikov, Alexander Alodjants, Anastasiia Trofimova, Dmitry Tsarev

**Affiliations:** 1 Mechanics and Optics (ITMO) Department, National Research University for Information Technology, 197101 St. Petersburg, Russia; 2 International Center for Mathematical Modeling in Physics and Cognitive Sciences, Linnaeus University, SE-351 95 Växjö, Sweden

**Keywords:** quantum-like models, operational approach, information interpretation of quantum theory, social laser, social energy, quantum information field, social atom, Bose–Einstein statistics, bandwagon effect, social thermodynamics, resonator of social laser, master equation for socio-information excitations

## Abstract

The recent years were characterized by increasing interest to applications of the quantum formalism outside physics, e.g., in psychology, decision-making, socio-political studies. To distinguish such approach from quantum physics, it is called *quantum-like.* It is applied to modeling socio-political processes on the basis of the social laser model describing *stimulated amplification of social actions.* The main aim of this paper is establishing the socio-psychological interpretations of the quantum notions playing the basic role in lasing modeling. By using the Copenhagen interpretation and the operational approach to the quantum formalism, we analyze the notion of the social energy. Quantum formalizations of such notions as a social atom, *s*-atom, and an information field are presented. The operational approach based on the creation and annihilation operators is used. We also introduce the notion of the social color of information excitations representing characteristics linked to lasing coherence of the type of collimation. The Bose–Einstein statistics of excitations is coupled with the *bandwagon effect,* one of the basic effects of social psychology. By using the operational interpretation of the social energy, we present the thermodynamical derivation of this quantum statistics. The crucial role of information overload generated by the modern mass-media is emphasized. In physics laser’s resonator, the optical cavity, plays the crucial role in amplification. We model the functioning of social laser’s resonator by “distilling” the physical scheme from connection with optics. As the mathematical basis, we use the master equation for the density operator for the quantum information field.

## 1. Introduction

From the very beginning, it has to be pointed out that we tried to make this paper readable for people working in psychology, decision-making, cognitive, social, and political science, and having minimal knowledge about the mathematical apparatus of quantum physics. Therefore, we try to minimize the number of mathematical expressions, except Section 4.4 and Section 9. The introduction is very detailed and its aim is to describe the general state of the art in applications of quantum theory to humanities. One one hand, we want to convince experts in humanities that quantum theory can resolve the well known problems, in particular, in decision theory. On the other hand, we want to convince physicists and especially those who work in quantum foundations and quantum information and probability that applications of quantum theory to humanities are not an exoticities: many top level experts, e.g., psychologists, work actively on quantum-like modeling.

### 1.1. Quantum versus Quantum-Like Models

Since the first days of quantum mechanics, the analogy between quantum physical and psychical processes sporadically attracted attention of leading physicists, psychologists, and philosophers. There can be mentioned the Pauli–Jung correspondence [1], see also [2], and Whitehead’s attempt to unify physical and mental processes within the quantum picture [3,4]. There should be also emphasized the numerous contributions to creation of quantum models of brain’s functioning [5,6,7,8,9,10,11,12,13,14,15]. However, we point out that the topic of this paper has nothing to do with their attempts to model cognition and consciousness from the genuine quantum physical processes in the brain. In the present paper, we proceed with the quantum-like approach, the operational application of the mathematical formalism of quantum theory, especially quantum probability and information, outside physics.

### 1.2. Quantum-Like Modeling of the Process of Decision-Making

Nowadays, quantum-like modeling is widely used in mathematical modeling in cognitive science, psychology, decision-making, game theory, economics and finances, social and political sciences (see, for example, monographs [16,17,18,19,20,21,22,23] and a few representative papers [24,25,26,27,28,29,30,31,32,33,34,35,36,37,38]). These applications are typically based on the use of quantum probabilistic calculus, instead of the classical one. It was demonstrated that experimental statistical data collected in, cognitive psychology, decision-making, social science, game theory, molecular biology, and epigenetic demonstrates quantum probabilistic features [18,19,22,23]. For example, such data can violate the classical formula of total probability and this violation can be mathematically expressed in the form of quantum interference. Some cognitive experiments demonstrating violation of the Bell type inequalities have been performed [35,36,37,38]. The violation can be interpreted as *contextuality of mental observables* represented as questions or tasks.

In theoretical studies in cognitive psychology and decision-making, quantum probability provides the adequate mathematical models for the basic psychological effects such as conjunction, disjunction, and order effects. Its use also resolves the fundamental paradoxes of the decision theory such as Allais, Ellsberg, and Machina paradoxes [39,40,41]. We remark that this resolution of the paradoxes of the classical decision theory (including expected utility, subjective utility, and prospect theories) and creation of the paradox-free (at least up to now) decision theory played a very important role in justification of applications of quantum probability outside physics.

### 1.3. Operational Formalism: Creation and Annihilation Operators

We emphasize that the quantum formalism provides only a formal operational description of physical processes; in particular, the spontaneous emission and stimulated absorption and emission which play the fundamental role in lasing theory. One of the important mathematical representations of the operational formalism is given in terms of the *creation and annihilation operators* ${\hat{a}}^{★},\hat{a}.$ In quantum optics, ${\hat{a}}^{★}$ represents creation of a photon through its emission; $\hat{a}$ represents disappearance of a photon from the field resulting from absorption of this photon by an atom. These are linear operators acting in complex Hilbert space representing the states of the quantum field. The operator ${\hat{a}}^{★}$ is adjoint to the operator $\hat{a}.$ The operator $\hat{n}={\hat{a}}^{★}\hat{a}$ plays the important role in the quantum field theory. This is the operator of the photons number. Its eigenstates are states |n〉 of the quantum field with the fixed number *n* of photons. Similar operational description can be given for the processes of state transitions in atoms.

The operational formalism is so useful in quantum theory, since here one cannot construct a more detailed description in terms of classical-like variables, known as hidden variables. Therefore, one proceeds with the operational formalism, representing preparations of system and observations. In this paper, as well in papers cited in Section 1.2, the operational formalism, including creation and annihilation operators (see, especially, [20,34]), is used to model the process of social lasing, stimulated amplification of social actions.

One of the reasons for application of the quantum operational formalism to modeling social processes is that, similarly to quantum physics, it is (practically) impossible to present the detailed account of all socio-psychological factors involved in stimulated amplification of social actions. In this situation, the operational description in terms of formal absorption and emission of information excitations can be fruitful.

We can refer to stormy discussions in socio-political literature (see [42,43,44,45,46] and further references in [47,48,49]) on the origin of color revolutions. These discussions are characterized by the diversity of opinions and the impossibility to present the detailed account of interrelation of all social, psychological, political, and economical and financial factors leading to such protest waves. The operational quantum model of “social laser” is based on ignoring these factors (playing the role of hidden variables). We are not interested in difference in, e.g., the emotional states of people in different countries or difference in the political situations in these countries. The operational formalism describes formally the processes of absorption and emission of portions of social energy; see Section 1.5 and Section 4.4 for further justification (from the information-theoretical and thermodynamical principles) of applying the quantum formalism to modeling of social lasing.

Nowadays, the big-data approach is widely applied for analysis of social processes. The deep learning is one of the cornerstones of the big-data project. We remark that the deep learning can be considered as a form of the operational description. The intermediate layers of networks performing deep learning are just the operational components having no real social or cognitive meaning. In contrast to the big-data approach, which is based on extensive consumption of computational resources, the quantum operational approach is endowed with the powerful mathematical formalism of analytic analysis. This formalism can be used for determination of the key-parameters playing the crucial role in stimulated amplification of social actions (see Section 9).

### 1.4. Social Laser as a Fruit of the Quantum Information Revolution

In this paper, we are interested in the information component of quantum theory. We recall that the recent quantum information revolution generated *the information interpretation of the quantum theory.* In fact, there were proposed a few often competing information interpretations [50,51,52,53,54,55,56,57,58,59,60,61,62,63]. However, all of them are characterized by the paradigm shift: quantum systems are treated as merely carriers of information. Thus, from this viewpoint, quantum theory is about information flows. Hence, it may be possible to apply the quantum information approach even outside physics by identifying the quantum-like features of processes under consideration.

In [47,48], the author proposed the quantum-like model of the social (or more generally information) laser, the social analog of the physical laser. The social laser theory was used for the mathematical description of *stimulated amplification of social actions* [49].

The model of the social laser is based on treatment of humans as carriers of *social energy*. In [47,48], the notion of the social energy was (rather schematically) discussed in the relation to the social lasing model. In this paper, we shall present the detailed analysis of this notion based on the operational approach to quantum theory and its information interpretation (Section 3).

In the simplest setting, we consider a two level cognitive system with the relaxation and excitation states. We call such a system a (two level) *social atom*, *s*-atom. In this paper, we restrict our considerations to two energy level systems. In the quantum-like modeling, the discrete structure of the social energy levels of *s*-atoms, energy quantization, has no straightforward relation to neurophysiology. In particular, the two levels structure is based on so to say “to be or not to be” scaling of the social energy. This is a kind of emotional quantity. Of course, emotions are coupled to physical and neurophysiological processes in human’s body, but this coupling is very complicated. In addition, it is not important for us in the present paper.

A social gain medium is composed of *s*-atoms. They interact with the information field generated by the mass-media and the Internet. This field is modeled as a quantum information field carrying quantized portions of social energy, social excitations. This field was formally introduced in [47,48]. Now, we put essential efforts to propose the proper interpretation of excitations of this quantum field as carriers of the social energy (Section 3.2).

Additionally to the social energy, we introduce the notion of a social color of an information excitation (Section 4.2). This quantity represents additional characteristics of information excitations linked to lasing coherence of the type of polarization and collimation (Section 4.5). We couple the *bandwagon effect,* one of the fundamental effects of social psychology, with the Bose–Einstein statistics of indistinguishable social excitations (Section 5).

### 1.5. Powerful Information Flows as the Basic Condition of Social Laser Functioning

The basic feature of quantum information fields is indistinguishability of excitations carried by them. This is indistinguishability with respect to the social energy carried by excitations (see Section 4.3 for further consideration).

The Bose–Einstein statistics of information excitations playing the crucial role in social lasing can be derived (by appealing to thermodynamical considerations) from such energy based indistinguishability; see Section 4.4.

People overloaded by information do not put essential efforts to analyze contents of communications delivered the by mass-media. They function as absorbers of social energy delivered by communications emitted by TV, newspapers, and the Internet. In particular, to approach population inversion, the mass-media can pump into a human gain medium a flow of shock news about various catastrophes, violence, and political scandals. Agents absorb the social energy. Later, this energy can be liberated and directed through the laser-like process of stimulated emission. Therefore, *the power of information flows plays the crucial role in creation of quantum-like processes of absorption and emission of the social energy.* Nowadays, this flow is extremely powerful. Hence, it is easier to create social (information) lasers than say 150 years ago.

### 1.6. Resonators of Physical and Social Lasers

In physics, the laser’s resonator in the form of the optical cavity plays the crucial role in the process of amplification of the output beam and making it coherent. In this paper, we model the functioning of social laser’s resonator by “distilling” the physical scheme to exclude the straightforward connection with optcis. We proceed with the *quantum master equation for the density operator* describing excitations of the quantum information field inside the resonator of the social laser; see Section 9.3. The main aim is establishing the proper social interpretations of the basic quantities and parameters of this dynamical system.

The Internet based *Echo Chamber* is considered as the important example of social resonators. Its functioning is mathematically represented by the field of social excitations in the form of posts and comments interacting with the human gain medium.

One of the basic consequence of the quantum-like dynamics is the existence of the threshold value for the pump parameter (see Section 9.5): if the power of pumping is essentially higher than this threshold, then practically all social energy pumped into the human gain medium is transferred into the output beam of social actions.

This is the good place to emphasize once again the role of huge power of the information flows generated by modern mass-media.

## 2. Physical Laser: Schematic Presentation

Since we really hope that this paper would be interesting for researchers working in cognitive, social, and political sciences and having no educational background in quantum physics, we present schematically the basic scheme of the physical laser functioning. For the moment, we do not consider the optical cavity component of the physical laser.

### 2.1. Spontaneous and Stimulated Emission

We start with description of the processes of spontaneous and stimulated emission for excited two level atoms with energy levels $E=E_{1},E_{2}.$ Denote the resonance energy of an atom by $E_{A}=E_{2}-E_{1}.$ Typically, in the physical literature, one proceeds with frequencies and speaks about the resonance frequency $\omega_{A}=\frac{E_{A}}{2\pi h}$, where *h* is the Planck constant. To proceed to the social modeling, we try to eliminate the space-time picture from the model. In particular, we want to operate solely with energies.

We consider the quantum electromagnetic field interacting with atoms and describe the physical processes generated by the interaction:

1. An atom in the excited state can spontaneously emit a photon. This process is irreducibly random, i.e., even for a single atom, it is impossible to predict neither the instance of time nor the direction of emitted photon.
2. Atoms in the ground state can absorb from this field only photons having the resonance energy $E_{A}=E_{2}-E_{1}.$
3. An excited atom interacting with photons with energy $E_{A}$ emits a photon of the same energy
4. This output flow of photons is coherent. All photons produced from the “seed-photon” have the same features: direction of flow, polarization, and energy.

**Figure 1:**
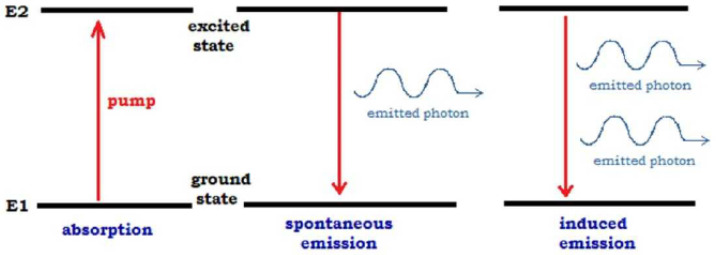
Emission and absorption of photons.

Thus, one photon produces two, these two interact with two atoms and produce four photons, after *n*-step, this process generates $N=2^{n}$ photons. We note that this description and its illustration by Figure 1, although typical for physics textbooks, is too straightforward. In fact, an atom interacts not with a single photon, but with all photons in the field (see Section 5 and Equation (10)).

### 2.2. Population Inversion

The cascade process described above plays the crucial role in generating laser beams carrying huge energy. One of the problems is spontaneous emission. (Of course, the primary problem is that the thermodynamic heat-bath Boltzmann distribution leads to higher population in the lower levels than in the upper levels.)

Pumping photons into a gain medium (an ensemble of atoms in the ground state) transfers ground state atoms into excited atoms. However, spontaneously, they can fall back to the ground level. The basic step in generating lasing is approaching *population inversion*. This is transition from an ensemble of atoms in the ground state to an ensemble in which more than 50% of atoms are in the excited state.

We remark that, for a gain medium composed of two level atoms, population inversion cannot be approached (at least straightforwardly) because the transition probabilities are equal: $p\left(E_{1}\to E_{2}\right)=p\left(E_{2}\to E_{1}\right).$ These probabilities are known as the *Einstein coefficients.* Their equality for the electromagnetic field can be proven by using thermodynamics for indistinguishable systems following Bose–Einstein statistics. For the electromagnetic field, one needs a gain medium composed of atoms with at least three energy levels, $E_{1}<E_{2}<E_{3}.$

The above considerations are about the physical laser based on the quantum electromagnetic field. However, in general, information thermodynamics [47] does not imply the coincidence of the Einstein coefficients for a two-level system. In principle, the social laser can be based on the simplest *s*-atoms having two levels of social energy.

## 3. Social Energy

The notion of *social energy* plays the crucial role in our framework. We start considerations with a general remark that in quantum theory value *a* of observable *A* obtained in its measurement cannot be interpreted as the property of system *S* on which the measurement is performed. By the Copenhagen interpretation, this value is generated in the complex process of interaction of *S* and a device used for *A*-measurement. In particular, this interpretation has to be applied to energy observable E. Although one often says, e.g., “the energy of the electron”, the correct meaning of this statement is about the output of the *E*-measurement. The Copenhagen interpretation is well accommodated to the notion of states’ *superposition.* Quantum system *S* can be in state ψ of superposition of two different energy levels $E_{1},E_{2}$

(1) $$\psi =c_{1}|E_{1}\rangle +c_{2}|E_{2}\rangle ,$$

where $c_{i}$ are complex numbers, probability amplitudes, such that $|c_{1}{|}^{2}+{\left|c_{2}\right|}^{2}=1$. If system *S* is in this state, then the probability to get the value $E_{i}$ of the energy observable is equal to $p_{i}={\left|c_{i}\right|}^{2}$. The states $|E_{1}\rangle ,|E_{2}\rangle$ correspond to the definite energy levels.

Supported by the quantum interpretation of the notion of energy, we are ready to consider the very complex notion of a *social (mental) energy.* This notion has been actively used in cognition, psychology, social and political science (since the works of James [64] and Freud [65,66], later by Jung [67], see also [68]) and recently in economics and finances, multi-agent modeling, evolution theory and industrial dynamics [69,70,71], see also [47,48,49] for details. In addition, of course, these previous studies are supporting for our model. However, we emphasize once again that the application of the Copenhagen methodology simplifies and clarifies essentially the issue of the social energy.

### 3.1. Energy of *s*-Atoms

In this framework, the simplest quantification of the social (mental) energy can be done by the question (observable) E= “Are you in the state of relaxation or excitement?” This observable takes two values, say $E_{1}=0,E_{2}=1.$ The Copenhagen interpretation is strongly involved. Before being asked the *E*-question, *s*-atoms can be in superposition of these two states. Only by confronting the *E*-question *s*-atom determines his/her state. The social energy observable can be represented in different forms; for example, in the form of the question E= “Shall you go to demonstration against Trump or Brexit?” Of course, we need not be restricted to the simplest “yes”–“no”, to be or not to be, quantification.

Finer quantifications of the social energy can considered as well. Different types of *s*-atoms can emit and absorb social energy portions of different magnitudes. Here, a type of an *s*-atom is determined by her/his psyche and social environment. In the operational formalism, we can proceed with some grading of the possible social energy levels for *s*-atoms. Since we restrict consideration to two-level *s*-atoms, they are characterized by the social energy levels $E=E_{1},E_{2},$ the ground and excited states, and the resonance energy $E_{A}=E_{2}-E_{1}.$ We remark that in principle the ground level energy for one type of *s*-atoms can be higher than the excitation level energy for another type. Social lasing is possible only for a gain medium with the homogeneous structure of energy levels.

This methodology demystifies the notion of social (mental) energy. Of course, the same Copenhagen methodology can be applied to any social (mental) observable. One should not be surprised that the methodology of quantum physics is applicable outside it. The Copenhagen interpretation presents the very general methodology which is applicable to any kind of measurement. We remark that the use of this measurement methodology does not imply that the whole apparatus of quantum theory can be applicable. One should be careful by checking constraints on the class of systems and observables leading to applicability of one or another part of quantum theory. The quantum-like approach does not mean to copy straightforwardly the complete quantum theory to say social science. For example, to derive the Bose–Einstein statistics, we have to assume indistinguishability of information quanta, excitations of the quantum information field (see Section 4.1).

### 3.2. Energy of the Quantum Information Field

In physics, energy can be assigned not only to atoms, material systems, but also to carriers of interactions, e.g., photons or neutrino, which are excitations of corresponding quantum fields. (Here “assigned” has the operational meaning: “can be measured”). In social lasing, the interaction processes are formally modeled with the aid of a quantum information field generated by a variety of information sources (see Section 4.1). Communications “emitted” by them carry portions of the social energy. These quanta of social energy are interpreted as excitations of the quantum information field.

Again, as in the case of *s*-atoms, we can proceed with “to be or not to be” quantification of the social energy carried by communications. If an *s*-atom in the ground state absorbs energy from communication C (and becomes excited), then C carries social energy $E_{2}=1.$ If C cannot excite a social atom, then C’s energy $E_{1}=0.$

This social energy quantification depends on the concrete ensemble of *s*-atoms, a social group. It is easy to give examples of social and political communications which would excite average Englishman or American, but not Russian or Chinese, and vice versa. Thus, the definition of information field’s energy is purely operational. In some sense, it is even “more operational” than the definition of the energy for a quantum physical field. It is meaningless to speak about the social energy of the information field without to describe the class of “detectors”; in our case, these are *s*-atoms. As in the case of *s*-atoms, it is possible to proceed with models based on finer scales of the social energy assigned to excitations of the information field. Each communication C is characterized by social energy $E_{C}.$ It can be absorbed by an *s*-atom with the resonance energy $E_{A}=E_{C}.$

A variety of communications can carry the same portion of the social energy. All communications with $E_{C}=E,$ where *E* is the fixed portion of the social energy are considered as equivalent from the viewpoint of energy delivering. They can be represented by the same field’s state |E〉, the ket-vector in field’s state space. We say that *E* determines *a mode of the quantum information field:* *E* is the analog of characteristic energy $E_{\omega }=h\omega$ of the electromagnetic mode with frequency ω.

How many elementary excitations of energy *E* are carried by the *E*-mode of the quantum information field? It depends on the power of information sources emitting communications belonging to the *E*-mode.

## 4. Social Laser

### 4.1. Quantum Field Representation of Information Flow Generated by Mass-Media

For the physical laser, the electromagnetic field is the basic energy source. The Bose–Einstein statistics of excitations (quanta) of this field plays the crucial role in laser’s functioning.

For a social analog of the physical laser, the basic (social) energy source is the *information field* generated by the mass-media and the Internet. As was discussed in Section 3.2, this field can be considered as composed of information quanta field’s excitations. These excitations are emitted by a variety of information channels and absorbed by humans, *s*-atoms. The information field is not a classical physical field defined on the physical space-time, as, e.g., the classical electromagnetic field given by the vector of the electric and magnetic fields $\left(E\left(x\right),B\left(x\right)\right),x\in R^{3}.$ We model the information field operationally with the aid of creation and annihilation operators, as a quantum field, see Section 1.3.

We remark that even in physics only the electromagnetic field has its classical counterpart with the space-time representation. If we consider, for example, the neutrino field, this field has only the quantum representation.

In the quantum field theory, a state of a field is mathematically described by a normalized vector belonging to the Fock space—the complex Hilbert space representing superpositions of possible excitations of the field. Consider the *E*-mode of the quantum information field. It represents excitations in the form of communications carrying the portion of social energy *E*: communications which are graded by the social group under consideration as having social energy E. In the Fock space representation, the quantum state of this field is represented as superposition

(2) $$\Phi =\sum c_{n}|n,E\rangle =\sum_{n}\frac{c_{n}}{\sqrt{n!}}{\hat{a}}_{E}^{★n}|0\rangle ,$$

where $\sum |c_{n}{|}^{2}=1.$ Here, ${\hat{a}}_{E}^{★}$ is the creation operator for *E*-excitations; |0〉 denotes the vacuum state of the field—no information excitations. For the real information sources, the sum is always finite. The use of infinite series is the price for mathematical consistency—the possibility to use a Hilbert space.

Generally, a social group is exposed to radiation of the quantum information field containing different modes of the social energy. It is natural to consider the discrete grading of the social energy. Here, we follow von Neumann who pointed out [72] that quantum observables with continuous spectrum are just mathematical idealizations of real measurement procedures. For such discrete grading, the Fock state can be written in the standard form

(3) $$\Phi =\sum \frac{c_{k}}{\sqrt{k!}}\;{\hat{a}}_{E_{1}}^{★k_{1}}\cdots {\hat{a}}_{E_{n}}^{★k_{n}}\cdots |0\rangle ,$$

where $k=(k_{j})$ and, for each multi-index k, only a finite number of $k_{j}$ differs from zero; the squared sum of absolute values of the coefficients equals to one.

### 4.2. Coloring Information Excitations

For the quantum electromagnetic field, excitations are photons and photon’s type is determined by index λ=(E,α). Here, *E* is photon’s energy and α encodes additional characteristics linked to lasing coherence of the type of polarization and collimation. In the same way, for the quantum information field, the type of an excitation is determined by index λ=(E,α). Here, *E* is *the social energy* carried by an excitation of the information field and α is a *social color* of this excitation encoding its basic social characteristics. The color of an information excitation is linked to coherence of social actions generated by a social laser. Thus, creation and annihilation operators have to be labeled not by just the energy index E, but by index λ, including the state representation, cf. Equation (3): $|n,\lambda \rangle ={\hat{a}}_{\lambda }^{★n}|0\rangle .$

We can mention a few examples of social colors of excitations of the quantum information field: war in Iraq, elections in USA, Brexit, tsunami in Japan, sex scandal, anti-globalism, climate change, rasism, sexism, Trump, and so on. Determination of social colors is a socio-psychological phenomenon. It depends on a social group. Thus social colors are not internal characteristics of the information field. Their depend on a social group exposed to “information radiation” generated by mass media.

The social analog of lasing can be initiated only in a social group, a social gain medium, with sufficiently rough coloring structure—to approach high concentration of excitations of the same social color mode in the *output beam* of excitations. For example, social color α= “sex scandal in Conservative and Unionist Party (UK)” is appropriate for social lasing. This is the proper social color for the communication: “A BOMBSHELL dossier naming and shaming 36 Tories suspected of inappropriate sexual behaviour has emerged as Westminster remains engulfed by a sex abuse scandal”. (Express, 30 October 2017). However, if somebody would spit this color and started to use 36 colors corresponding to concrete Tory-executives involved in the sex scandals, then such *s*-atom is not a proper subject for social lasing.

### 4.3. Indistinguishability from Information Overload and Complexity

For *s*-atoms, excitations of the information field carrying the same social energy and color, λ=(E,α), are *indistinguishable.* Indistinguishability is the basic condition leading to quantum statistics through thermodynamical analysis, both for physical and information systems (see Section 4.4). Indistinguishability of excitations generated by the mass-media is relative. It corresponds to coarse graining of coloring and depends on a social group, a collection of *s*-atoms. If *s*-atoms do not operate in the indistinguashability regime, i.e., they perform the detailed analysis of contents of communications, then the quantum statistical description is inapplicable for them. In this case, social lasing is impossible. We remark that the detailed analysis of communication’s content preassumes the use of Boolean logic. Thus, the indistinguishability regime implies deviations from Boolean logic, cf. [73,74].

The *information overload* is one of the basic reasons for operation at the indistinguishability regime. People simply do not have time and information processing resources for the deep analysis of communications’ contents. They proceed with coarse graining leading to relative indistinduishability. Here, “relative” has the meaning of relative with respect to a social group. In the modern human society, the information overload is combined with complexity of information delivered by a variety of information sources. The majority of population simply does not have mental capacity to perform the detailed analysis of complex socio-political, financial, and ecological problems.

Coarse graining, “rough coloring” and the indistinguishability regime are the basic features of human cognition. However, the information overload and complexity led to tremendous increase of their role.

### 4.4. From Statistical Mechanics to Thermodynamics of Indistinguishable Systems

Here, we follow [47], but with emphasis of the operational meaning of the notion of the social energy. In turn, the presentation in paper [47] on transition from statistical mechanics of indistinguishable systems to thermodynamics is based on Schrödinger’s book [75].

Consider a system which is composed of *m* indistinguishable subsystems. Compound system will be denoted by S and its subsystems by *S* with indexes. It is assumed that an observer can assign to all systems some quantity called energy and satisfying the additivity requirement with respect to its distribution over subsystems of a system. We shall denote the energy of S by E and the energy of *S* by *E*. These quantities should not be interpreted as objective properties of systems. Energy E=E(S) can be assigned to *S* as the output of observation performed by an observer on *S* The same interpretation is used for energy E=E(S) assigned to S.

As always derivation of thermodynamical quantities from ensemble statistics is started with construction of *partition function* Z. The possible energy levels of a subsystem *S* are denoted by $E_{1},\ldots ,E_{j},\ldots$. An energy level $E_{k}$ of *m*-particle compound system S is characterized by a sequence of natural numbers $m_{1},\ldots ,m_{j},\ldots$ of subsystems on the corresponding levels. The latter means “subsystems with measurement outputs” $E_{1},\ldots ,E_{j},\ldots$. Here, it is crucial that subsystems with the same value of energy $E_{j}$ are not distinguished one from another. This indistinguishability determines the form of *Z* and, hence, all thermodynamical quantities. We have

(4) $$E_{k}=\sum_{s}E_{s}m_{s}.$$

Thus, the partition function is given by the sum

(5) $$Z=\sum_{\left(m_{s}\right)}e^{-\mu \sum_{s}E_{s}m_{s}},$$

where symbol $\left(m_{s}\right)$ denotes an admissible set of numbers $m_{s}.$ For the moment, μ>0 is just a parameter of the model.

From lnZ, it is possible to deduce the basic thermodynamical quantities; in particular, the average value of $m_{s}$

(6) $${\bar{m}}_{s}=-\frac{1}{\mu }\frac{\partial \ln Z}{\partial E_{s}}.$$

Now, different statistics for systems composed of indistinguishable subsystems can be obtained by consideration of different possible ranges of values of natural numbers $m_{s}:$

1. $m_{s}=0,1,2,\ldots .$ (Bose–Einstein statistics),
2. $m_{s}=0,\ldots ,q,$ where q≥1 is a natural number (parastatistics).

In physics, q=1, i.e., $m_{s}=0,1;$ this is the case of the Fermi–Dirac statistics. We remark that in quantum physics selection of the Fermi–Dirac statistics from a bunch of parastistics is just postulated. Of course, it is confirmed by the experimental situation. One cannot exclude that in social science parastistics with q>1 can find applications.

By restricting considerations to the Bose–Einstein and Fermi–Dirac statistics and following Schrödinger [75], we find that the corresponding partition functions can be expressed as

(7) $$Z=Z_{\mathrm{BE}}=\prod_{s}\frac{1}{1-e^{-\mu E_{s}}},\;Z=Z_{\mathrm{FD}}=\prod_{s}\left(1+e^{-\mu E_{s}}\right).$$

This leads to the following basic expression for the average value of $m_{s}$

(8) $${\bar{m}}_{s}=\frac{1}{\frac{1}{\xi }e^{\mu E_{s}}\mp 1},$$

where 0<ξ≤1 is so called parameter of degeneration, ξ=ξ(m). We remark that, for photons, ξ=1, with the Bose–Einstein statistics.

Then, one gets the average energy as

(9) $$U=\sum_{s}\frac{\alpha_{s}}{\frac{1}{\xi }e^{\mu E_{s}}\mp 1}.$$

In physics, the quantity *T* inverse to parameter μ is interpreted as temperature. In social modeling, we can speak about a kind of the *social temperature.* This is a complicated notion and we are not ready to discuss it in detail in this paper. On one hand, we can try to proceed as in classical thermodynamics. However, even by mimicking classical thermodynamics, it is important to remember that such a quantity is of the socio-emotional type. Thus, it cannot be considered as the objective feature of a social system. One can try to define social temperature through consideration of classes of equivalent thermometers, measurement procedures for the social temperature. However, the above thermodynamical considerations for indistinguishable systems represent the quantum situation. Hence, the classical definition of temperature does not match them. As in quantum physics, we can try to introduce a kind of social temperature as a parameter characterizing phase transitions. This is a complicated mathematical theory and we postpone such considerations to one of further publications.

### 4.5. Coloring Role: Pumping versus Emission

We also point to the following striking similarity in behavior of atoms and *s*-atoms. A portion of social energy absorbed by *s*-atom generally “lost its color”.

Somebody, say Elena, living in Moscow absorbed a social excitation emitted by the Russian radio-station, *Echo of Moscow.* Typically, such excitations have the anti-corruption colors. However, this does not mean that her spontaneous relaxation would be directed against corruption. She can emit the portion of social energy absorbed from *Echo of Moscow* in a family scandal or another kind of private or social action.

Such behavior is similar to behavior of atoms interacting with photons. If an atom absorbs a photon carrying some concrete physical characteristics, “color”, such as direction or polarization, then it immediately forgets about its pre-absorption value. Later, it can emit a photon with a different color, i.e., a photon flying in the direction different from the direction of the pre-absorption photon.

However, we remind that the quantum theory is about observational quantities. We do not know what happens inside the atom between absorption and emission of photons. We neither know what happens inside the head between hearing the news from *Echo of Moscow* and going to the kitchen to start scandaling or to the center of Moscow to protest.

This kind of memory lost can be very useful in approaching population inversion (see [47]). There is no need to pump in a gain medium the social energy of the same color as in laser’s output beam. In principle, the social energy pumping need not be based solely on information about corruption and other dysfunctions of the government. Shocking news about catastrophes, tornado, killers are the important part of energy-pumping in human gain medias.

Now, we turn to physics. In contrast to the process of absorption-emission, the process of stimulated emission of photons is characterized by “color” conservation: the “colors” of the emitted photon and photons stimulating emission coincide. We also point out that in the process of stimulated emission, the crucial role is played by intensity of the stimulating electromagnetic field, so-called Bosonic effect: increase of probability of stimulated emission with increase of intensity of the flow bosonic excitations (see Section 5 and Equation (10)).

In Section 5, we compare this effect with the bandwagon effect in psychology. The operational identity of these two effects, physical and psychological, supports application of the quantum field formalism and methodology to social processes. Stimulated emission of social excitations by excited *s*-atoms can be considered as exhibition of the bandwagon effect: *s*-atom in the excited state exposed by radiation compounded of α-colored social excitations would emit a social excitations of the same color.

## 5. Comparing Stimulated Emission in Quantum Physics and Bandwagon Effect in Psychology and Social Science

We stress that the quantum field description of the stimulated emission is a *collective effect*, i.e., an atom interacts with a bunch of photons and not just with an individual photon. It interacts with all excitations of the quantum electromagnetic field having the resonance energy of this atom, $E_{A}=E_{2}-E_{1}.$

*The crucial role is played by the Bose–Einstein statistics of the photons.* We consider the fixed energy (frequency) mode of the quantum electromagnetic field. For fixed color mode α, *n*-photon state |n,α〉, can be represented in the form of the action of the photon creation operator $a_{\alpha }^{★}$ corresponding to this mode on the vacuum state |0〉:

(10) $$|n,\alpha \rangle =\left[{\left(a_{\alpha }^{★}\right)}^{n}/\sqrt{n!}\right]|0\rangle .$$

This representation gives the possibility to find that the transition probability amplitude from the state |n,α〉 to the state |n+1,α〉 equals to $\sqrt{\left(n+1\right)}.$ On the other hand, it is well known that the reverse process of absorption characterized by the transition probability amplitude from the state |n,α〉 to the state |(n−1),α〉 equals to $\sqrt{n}.$

Thus, in a quantum Bosonic field increasing photons’ number leads to increasing the probability of generation of one more photon in the same state. This constitutes one of the basic quantum advantages of laser stimulated emission showing that the emission of a coherent photon is more probable than the absorption. This is the strong argument in favor of using the quantum modeling of lasing.

This behavior of photons or more generally excitations of any quantum Bosonic field matches *the cognitive bias known as the bandwagon effect* [76]. This effect is characterized by the probability of individual adoption increasing with respect to the proportion who have already done so. People are not interested in underlying rational justification based on Boolean logic. They “hop on the bandwagon” by taking into account only the number *n* of those who are already seating on it. It is important to stress that, for an agent interacting with bandwagon’s population, personalities of people on bandwagon play no role: these people are indistinguishable.

This indistinguishability is only with respect to this concrete interaction: social, financial, racial, gender, or political action characterizing this “bandwagon.” Of course, people seating on a bandwagon are individual agents who can differ crucially: biologically, mentally, culturally.

As we have already emphasized, indistinguishability is the crucial assumption leading to quantum statistics. In the case of bandwagon effect, this is the Bose–Einstein statistics. Of course, it need not be exactly the photon statistics.

*Thus, the bandwagon effect can be considered as social exhibition of the Bose–Einstein statistics caused by indistinguishability.*

To model social lasing, we consider the information version of the bandwagon effect: *s*-atom interacts mainly not with other *s*-atoms, but with excitations of the information field generated by the mass-media and the Internet as well as emitted by other agents. For the concrete social color, these excitations are indistinguishable. In addition, *s*-atom in the excited state surrounded by information excitations of social color α emits excitation of the same color with probability proportional to the number of excitations. The crucial role is played by the field coherence, with respect to the concrete color.

We can conclude that the formal mathematical model is the same for physical and social structures. This is the model of stimulated emission of a system, physical or human, interacting with some Bosonic field.

## 6. Social Lasing Schematically

Each class of information communications is characterized by the social energy. Coherence corresponds to social colour sharpness (ideally one single mode α) generating a coherent beam of social actions. People in the excited state interacting with α-colored excitations of the information field would also emit α-colored excitations.

For example, a gain medium consisting of agents in the excited state and stimulated by the anti-corruption coloured information field would “radiate” a wave of anti-corruption protests. The same gain medium stimulated by some information field carrying another social colour would generate the wave of actions corresponding this last colour.

The amount of the social energy carried by communications stimulating lasing should match the resonance energy of *s*-atoms in the human gain medium.

To approach the population inversion, the social energy is pumped into the gain medium. This energy pumping is generated by the mass-media and the Internet sources. The gain medium should be homogeneous with respect to the social energy spectrum. In the ideal case, all *s*-atoms in the gain medium should have the same spectrum, $E=E_{1},E_{2}.$ In reality, it is impossible to create such a human gain medium. As in physics, the spectral line broadening has to be taken into account.

Social colors of excitations in the energy pumping beam have no straightforward connection with the social color of excitations in the output beam.

## 7. Resonators of Physical Lasers

In laser physics one of the main problems in lasing initiation is approaching the population inversion. However, population inversion is not enough to generate lasing. Stimulated and spontaneous emissions are competing with each other. Thus, before becoming an amplifying device, a gain medium pumped by an external energy source is first radiated as a usual electric “lamp”. Here, spontaneous emission is dominating. The light power is distributed over a variety of frequencies and directions of propagation, generally uniformly distributed. It is the optical cavity, laser’s resonator, that creates the conditions necessary for stimulated emission to become predominant over spontaneous emission.

*In further considerations, it is assumed that the gain medium has approached population inversion.*

The cavity or resonator is composed of two mirrors that *bounce the beam back and forth through the gain medium.* The cascade process of increasing photons’ number inside the cavity can be initiated either by spontaneous emission from an atom in the gain medium or by a bunch of photons injected in the optical cavity. (The photons carry the energy-quanta matching the energy levels of atoms in the gain medium.) In the latter case, these photons interact with atoms and generate stimulated emission. It is crucial that these photons have the same phase. One can imagine them as a cloud of exponentially increasing size moving between mirrors. *The concentration of the field inside the cavity increases the probability of stimulated emission rather than spontaneous emission occurring. This is the basic feature of bosons* (see Section 5). We repeat that *bosons’ behavior is similar to human’s behavior known as the bandwagon effect* (see again Section 5).

## 8. Resonators of Social Lasers

The same competition between spontaneous and stimulated emission plays the crucial role in social processes. People in the excited state may “radiate” social energy spontaneously, say in debates with relatives and friends about the political and social problems. Social colors of excitations in such spontaneous radiation are typically randomly distributed, often uniformly distributed. Such emission of social energy cannot lead to coherent social actions.

### 8.1. Structure and Functioning of Social Resonator

A social resonator consists of a gain medium composed of *s*-atoms which has already approached population inversion and say an Internet based communication system, e.g., some social network. We call such a system *Echo Chamber.* We restrict modeling to the Internet based Echo Chambers. Consider the following idealized model.

Each *s*-atom in the excited state can emit a quantum of social energy in the form of a post or a comment on some post. We call them, posts and comments, *excitations of the social resonator.* By posting or commenting, i.e., emitting an excitation, *s*-atom falls to the ground state. Resonator’s excitations play the role of photons in the optical cavity, the resonator of the physical laser. Moreover, to simplify the model, we assume that the social resonator under consideration accepts only excitations of the concrete color α. This is the strong constraint that is in visible contradiction with functioning of typical Internet based social networks. We shall relax it in later modeling. The social color of an excitation plays the role of the direction of propagation of output beam of photons emitted by laser’s resonator, the *x*-axis of the optical cavity.

Suppose that, at the fixed instance of time in the social resonator, there are *n* excitations. Each member of the gain medium interacts with all these excitations—with the information field. The boson behavior of excitations implies that the probability that the concrete agent would fall to the ground state and emit an excitation increases with *n*, Section 5. It is crucial that, if all excitations of the social resonator have fixed color α, the color of excitation emitted by this agent is also α. These dynamics lead to the exponential increasing the number *n* of excitations having the α-color inside this social resonator (see Section 9 for modeling of temporal dynamics). Excitations of colors different from α also can be spontaneously emitted by the gain medium. However, they cannot generate the cascade process, since in the present model they are simply blocked.

#### Output Beam from Echo Chamber

When *n* becomes sufficiently large, see Section 9, it is possible to open the output channel of the Echo Chamber and generate the stable flow of high intensity of excitations of the fixed α-color. In “outer space”, this flow is realized in the form of meetings, demonstrations, and brutal protest actions.

### 8.2. Stimulated Initiation of Social Lasing

As was stressed, the straightforward blocking of excitations with colors different from one fixed color α is the strong assumption. Of course, moderators of social networks block some posts and comments, e.g., having extremist, racist, or sexist content. However, the proportion of filtered excitations seems not to be so high, in any event it is far from 100%. Therefore, we have to improve the above model. As was presented in Section 7, there are two possible scenarios for initiating lasing:

1. spontaneous emission and filtering photons with momentum vectors deviating from the *x*-axis by using the optical cavity;
2. stimulated emission generated by a coherently injected ensemble of photons with the *x*-momentum vector.

In social lasing, the second scenario is preferable because the social mechanism of *filtering* of “wrongly directed and spontaneously emitted social excitations” is not so straightforward as in optics. Actually, the components which are not coherent with the beam are not eliminated, but become insignificant and can be considered as “noise”, as if they were in some way “ignored” by the mainstream social movement.

Thus, generation of the beam of social excitations having the same color α is started by injection a block of α-colored posts into the Internet Echo Chamber. They are injected in the same moment of time. (Of course, one has to take into account the temporal scale of Echo Chamber’s functioning). This initializing block of excitations generates the cascade of stimulated emissions. After a few interactions, the propagating wave of excitations is so big that the probability of stimulated emission becomes very close to one. This is a good time to open the output channel of the Echo Chamber and to transform information excitations into physical social actions.

Of course, spontaneously posted excitations of colors different from α can also be generated in this Echo Chamber. However, they are generated in different moments and have a variety of colors. Even if such a post starts to generate its own cascade, its power is negligible comparing with the dominating cascade started with injection of α-posts.

## 9. Dynamics the Quantum Information Field in Social Laser’s Resonator

We proceed with the standard formalism of theory of open quantum systems by using the quantum master equation for the state of a subsystem of a compound system. In our considerations, the latter consists of the quantum information field interacting with the *s*-atom gain medium. The basic dynamical equation is given by the quantum Markov approximation of the Schrödinger dynamics for the state of the compound system. This is the *Gorini–Kossakowski–Sudarshan–Lindblad* (GKSL) equation [77]. We point out that the using the Markov approximation is an important assumption. Its meaning and validity for social systems is a complex question (see [78] for the corresponding analysis of the general socio-political situation). Applicability of the quantum Markov approximation to modeling social lasing should be studied in more detail. We have no possibility to do this in the present paper but plan to turn to this problem in one of the future works.

As always in theory of open quantum systems, we should extend the notion of the state of a social system by considering mixed states represented by density operators.

We follow presentations in physical works, e.g., [79,80,81,82,83]. One of the essential differences in transition from quantum modeling of real physical processes to quantum-like modeling outside physics is that the Planck constant *h* cannot be considered as social action quantum. In quantum physics, the constant *h* couples photon’s energy *E* and angular frequency ω as

(11) E=ℏω,

where ℏ=h/2π is the reduced Planck constant. As was already mentioned, we were not able to find a natural social analog of angular frequency ω. We tried to proceed without it. Equation (11) can be written as

(12) τ=1/ω=ℏ/E.

Here, τ has the physical dimension of time and it can be interpreted as the time scale of the quantum dynamics for the *E*-mode of the quantum electromagnetic field.

By transition from genuine quantum to quantum-like modeling, we have to introduce an analog of the Planck constant, say γ. It is interpreted similarly to Equation (12), as a constant coupling the time scale and energy. There is no reason to assume that it is equal to the physical constant *ℏ*. Moreover, we cannot assume that γ is the same for all social processes. If it were the case, it would be really surprising! This is just the time scale of a social process modeled with the aid of the quantum formalism.

### 9.1. Creation-Annihilation Algebras for *s*-Atoms and Quantum Information Field

The quantum information field carrying the fixed amount of social energy $E_{F}$ can be represented in the following operator form

(13) $$\hat{E}\left(t\right)=ue^{-it\frac{E_{F}}{\gamma }}\hat{a}+\bar{u}e^{it\frac{E_{F}}{\gamma }}{\hat{a}}^{★},$$

where *u* is the complex field amplitude and $\hat{a}$ and ${\hat{a}}^{★}$ are annihilation and creation operators for social excitations of $\hat{E}\left(t\right),$ i.e.,

(14) $$\hat{a}|n\rangle =\sqrt{n}|n-1\rangle ,\;{\hat{a}}^{★}|n\rangle =\sqrt{n+1}|n+1\rangle .$$

Here, scaling constants $\sqrt{n},\sqrt{n+1}$ are selected in such a way that the operator

(15) $$\hat{n}={\hat{a}}^{★}\hat{a}$$

can be interpreted as the operator of the excitations’ number: $\hat{n}|n\rangle =n|n\rangle .$

The creation–annihilation operators satisfy the canonical commutation relation:

(16) $$\left[\hat{a},{\hat{a}}^{★}\right]=\hat{a}{\hat{a}}^{★}-{\hat{a}}^{★}\hat{a}=I,$$

where *I* is the unit operator.

The information field Hamiltonian is given by

(17) $${\hat{H}}_{F}=E_{F}\;\hat{n}.$$

The *n*-excitation state |n〉 is the eigenstate of the field Hamiltonian. In this state, the field energy equals $nE_{F}.$ In the absence of interactions, it is preserved in the process of field’s evolution.

As everywhere in this paper, we consider *s*-atoms with the two-level structure of the social energy, $E=E_{1},E_{2}$ and transition energy $E_{A}=E_{2}-E_{1}.$ Energy lowering and rising can be formally described by creation and annihilation operators. In contrast to the information field, these are fermionic operators:

(18) $$\hat{b}|E_{1}\rangle =0,\;{\hat{b}}^{★}|E_{1}\rangle =|E_{2}\rangle ,\;\hat{b}|E_{2}\rangle =|E_{1}\rangle ,\;{\hat{b}}^{★}|E_{2}\rangle =0.$$

Hence, they satisfy the fermionic canonical commutation relation:

(19) $$\left\{\hat{b},{\hat{b}}^{★}\right\}=\hat{b}{\hat{b}}^{★}+{\hat{b}}^{★}\hat{b}=I.$$

These operators are also known as *level lowering and rising operators.* Here, the vectors $|E_{1}\rangle$ and $|E_{2}\rangle$ represent the ground and excited states, respectively. Similarly to the field’s excitations number operator $\hat{n},$ we set ${\hat{n}}_{A}={\hat{b}}^{★}b.$ We have ${\hat{n}}_{A}|E_{1}\rangle =0$ and ${\hat{n}}_{A}|E_{2}\rangle =|E_{2}\rangle .$

We also note that the field and *s*-atom’s operators commute:

(20) $$\left[\hat{a},\hat{b}\right]=\left[\hat{a},{\hat{b}}^{★}\right]=\left[{\hat{a}}^{★},\hat{b}\right]=\left[{\hat{a}}^{★},{\hat{b}}^{★}\right]=0.$$

The reader should not be disappointed that to model transitions between *s*-atom’s states as well as information filed’s states, we use the same operator algebra as in physics. The formalism of creation and annihilation operators can be used in all models describing transitions between states. For example, besides quantum physics, this formalism plays the important role in analysis of reaction–diffusion equations. This formalism is widely used to model human cognition, decision-making, in finances, and political studies [20,29,34].

Hamiltonian of an *s*-atom is given by

(21) $${\hat{H}}_{A}=E_{1}+E_{A}{\hat{n}}_{A}.$$

Since ${\hat{H}}_{A}|E_{\alpha }\rangle =E_{\alpha }|E_{\alpha }\rangle ,\alpha =0,1,$ in this model, an *s*-atom who is isolated from the information field and being in the ground state cannot become excited by herself and being in the excited state cannot emit spontaneously a social excitation and relax.

In fact, the forms of the field and *s*-atom Hamiltonians are selected to preserve the states of the concrete social energy (in the absence of interactions). Thus, the forms of these Hamiltonians express the law of energy conservation, but only for the states of the concrete energy. The same energy conservation constraint is supposed in quantum physics. Therefore, the reader should not surprised that we proceed with Hamiltonians of the same form as in physics.

### 9.2. Dynamics of the Compound System *s*-Atom-Field

Interaction between the quantum information field and *s*-atoms is described by Hamiltonian

(22) $${\hat{H}}_{I}=-\gamma g\left(\hat{b}\left(t\right)+{\hat{b}}^{★}\left(t\right)\right)\hat{E}\left(t\right),$$

where the parameter g>0 expresses the strength of coupling between *s*-atoms and the information field.

The dynamics of *s*-atom’s creation and annihilation operators is given by

(23) $$\hat{b}\left(t\right)=e^{-it\frac{E_{A}}{\gamma }}\hat{b},\;\;{\hat{b}}^{★}\left(t\right)=e^{it\frac{E_{A}}{\gamma }}b^{★}.$$

This dynamics is just the Heisenberg picture of the state dynamics given by the Schrödinger equation.

Consider action of the *s*-atom component of the interaction Hamiltonian at t=0,

(24) $$\left(\hat{b}+{\hat{b}}^{★}\right)|E_{1}\rangle =|E_{2}\rangle ,\;\left(\hat{b}+{\hat{b}}^{★}\right)|E_{2}\rangle =|E_{1}\rangle .$$

This is the flipping-operator representing transitions between states of an *s*-atom. Such transitions should be compensated by modification of the information field state. Mathematically, this interaction process is represented as composition of two operators.

Formally, we should also use the time-dependent creation and annihilation operators in free Hamiltonians ${\hat{H}}_{A},{\hat{H}}_{F}.$ However, these Hamiltonians contain only compositions of operators of the forms ${\hat{b}}^{★}\left(t\right)\hat{b}\left(t\right),{\hat{a}}^{★}\left(t\right)\hat{a}\left(t\right)$ and complex exponents containing time dependence cancel each other.

Finally, the interaction Hamiltonian can be represented in the form:

(25) $${\hat{H}}_{I}=-\gamma g[ue^{-it\frac{E_{F}+E_{A}}{\gamma }}\hat{a}\hat{b}+\bar{u}e^{it\frac{E_{F}+E_{A}}{\gamma }}{\hat{a}}^{★}{\hat{b}}^{★}+$$

$$ue^{-it\frac{E_{F}-E_{A}}{\gamma }}\hat{a}{\hat{b}}^{★}+\bar{u}e^{it\frac{E_{F}-E_{A}}{\gamma }}{\hat{a}}^{★}\hat{b}].$$

The Hamiltonian of the compound system, the quantum information field carrying the social energy $E_{F}$ and interacting with an *s*-atom having the social energy spectrum $E=E_{1},E_{2},$ is given by

$$\hat{H}={\hat{H}}_{A}+{\hat{H}}_{F}+{\hat{H}}_{I}.$$

Denote by ρ the state of this compound system. Its dynamics is described by the von Neumann equation:

(26) $$i\gamma \frac{\partial \rho }{\partial t}\left(t\right)=\left[\hat{H},\rho \left(t\right)\right],$$

with the initial condition

(27) $$\rho \left(t_{0}\right)=\rho_{A}\left(t_{0}\right)⊗\rho_{F}\left(t_{0}\right).$$

Here, it is supposed that at $t=t_{0}$ *s*-atom’s and field’s information states are uncorrelated. Mathematically, this absence of correlations is represented by factorization of the compound system state into the states of the *s*-atom and the field.

As typical in physical considerations, it is convenient to move to the interaction representation. In this representation, the two components of dynamics, one given by Hamiltonian $H_{\mathrm{free}}={\hat{H}}_{A}+{\hat{H}}_{F}$ and another by interaction Hamiltonian $H_{I},$ are spit. The first part is used for transformation of the density operator ρ(t) of the form $\tilde{\rho }\left(t\right)=U^{★}\left(t\right)\rho \left(t\right)U\left(t\right),$ where U(t) is the unitary one parametric group describing the dynamics generated by $H_{\mathrm{free}},$ i.e., $U\left(t\right)=e^{-iH_{\mathrm{free}}/\gamma }.$ This is the transformation to the interaction representation. In addition, in the latter state’s dynamics is generated by $H_{I}.$

To simplify notations, in the interaction representation, we will use the symbol ρ(t) to denote the state (instead of symbol $\tilde{\rho }\left(t\right)).$ In this representation, the von Neumann equation has the form:

(28) $$i\gamma \frac{\partial \rho }{\partial t}\left(t\right)=\left[{\hat{H}}_{I},\rho \left(t\right)\right].$$

This equation can be solved approximately by iterated integration starting with

(29) $$\rho^{\left(1\right)}\left(t\right)=\rho \left(t_{0}\right)+\frac{1}{i\gamma }\int_{t_{0}}^{t}\left[{\hat{H}}_{I},\rho \left(s\right)\right]ds.$$

### 9.3. Gorini–Kossakowski–Sudarshan–Lindblad Equation for the State of the Quantum Information Field

By using the above integral iterations and under some assumptions (see Section 9.4), we can derive the approximate quantum master equation for the reduced density operator of the field,

$$\rho_{F}\left(t\right)\equiv {\mathrm{Tr}}_{A}æ\left(t\right),$$

where the partial trace is with respect to basis $|E_{1}\rangle ,|E_{2}\rangle$ in the state space of *s*-atom’s social energy. This is the special case of the quantum Markov approximation for the dynamics of a subsystem—of a compound quantum system described by the GKSL-equation.

Our main task is to present the proper social interpretations for the parameters of the GKSL-equation. For *s*-atoms composing the gain medium, denote by $T_{i},i=1,2,$ the average times of being in the ground and excited states, respectively. We can call them the lifetimes of relaxation and excitation. Denote by $r_{2}$ the rate of excitation of *s*-atoms generated by social energy pumping into this gain medium and by $r_{1}$ the absorption rate.

The quantum master equation has the form:

(30) $$\frac{\partial \rho_{F}}{\partial t}\left(t\right)=-A\left[\hat{a}{\hat{a}}^{★}\rho_{F}\left(t\right)+{\hat{a}}^{★}\rho_{F}\left(t\right)\hat{a}+h.c\right]+B\left[\hat{a}{\hat{a}}^{★}{æ}_{F}\left(t\right)+3\hat{a}{\hat{a}}^{★}{æ}_{F}\left(t\right)\hat{a}{\hat{a}}^{★}-4{\hat{a}}^{★}\hat{a}{\hat{a}}^{★}{æ}_{F}\left(t\right)\hat{a}+h.c\right]$$

$$-C[{\hat{a}}^{★}\hat{a}\rho_{F}\left(t\right)+\hat{a}\rho_{F}\left(t\right){\hat{a}}^{★}+h.c],$$

where h.c is the abbreviation for “Hermitian conjugate.” Here,

(31) $$A=r_{2}{\left(gT_{2}\right)}^{2}{\left|u\right|}^{2}$$

and

(32) $$C=r_{1}{\left(gT_{1}\right)}^{2}{\left|u\right|}^{2}$$

are *the gain and loss coefficients*, respectively. These coefficients depend quadratically on the amplitude of the information field u, the interaction coefficient g, and excitation and relaxation lifetimes $T_{2}$ and $T_{1}.$ They are linearly proportional to excitation and absorption rates, $r_{2},r_{1}.$ The term with the saturation coefficient

(33) $$B=r_{2}{\left(gT_{2}\right)}^{4}{\left|u\right|}^{4}$$

plays the crucial role in generation of exponential increase of the number of excitations in the field. We shall turn to this question later by considering the dynamics of probabilities.

We remark that, since in Equation (30) the operator coefficients are time-independent, the dynamical state update is not based on the long term memory. If we consider a discrete time approximation of this dynamics, $\rho_{F}\left(t_{k}\right),t_{k}=t_{0}+k\delta t,$ then the state at the moment $t_{k+1}$ is completely determined by the state at $t=t_{k}.$ Such property is known as the Markov property.

### 9.4. Social Interpretation of Assumptions for Derivation of Quantum Master Equation

There are two basic assumptions for derivation of master Equation (30) from von Neumann Equation (26) (see [83]). To formulate these two assumptions, it is convenient to introduce the time scale

(34) $$\tau_{F}=\frac{\gamma }{E_{F}}$$

of the evolution of field’s mode with the energy $E_{F}$ and the time scale

(35) $$\tau_{A}=\frac{\gamma }{E_{A}}$$

of transition between the states $|E_{i}\rangle ,i=1,2.$

**Assumption** **1.** *Analysis of dynamics (30) can be essentially simplified under the following condition:*

(36) $$2\pi \frac{\gamma }{E_{A}}<<T_{2}.$$

*In this situation, the first two terms in the expression (25) of the interaction Hamiltonian $H_{I}$ can be neglected in the process of integration with respect to time (see (29)).*

*Inequality (39) can be written as*

(37) $$\frac{T_{2}}{\tau_{A}}>>2\pi .$$

*Thus, the lifetime of the excited state of an s-atom should be long enough comparing with the transition time scale $\tau_{A}.$ We note that the latter is inversely proportional to the resonance energy $E_{A}=E_{2}-E_{1}.$*

*For a gain medium with a large gap of the social energy, it is easier to satisfy the condition (37). For such a social group, the master Equation (30) gives a better approximation. Of course, the condition (37) expresses the complex interplay between the magnitudes of the lifetime for the excited state $|E_{2}\rangle$ and the size of the energy gap $E_{A}.$*

*A good gain medium is characterized by the long lifetime of the excited state and the big gap between the states of relaxation and excitation.*

*For such a gain medium, the master equation approximation gives the adequate picture of social processes in the gain medium (under additional Assumption 2).*

**Assumption** **2.** *The difference between the energies $E_{A}$ and $E_{F}$ is very small, so the social energy of quanta associated with communications compounding the field differs not so much from the resonance energy of s-atoms—the social energy for transition between s-atoms’ states:*

(38) $$|E_{F}-E_{A}|\frac{T_{2}}{\gamma }<<1.$$

*This condition of matching social energies is natural for s-atoms interacting with the information field. The social energy $E_{F}$ carried by excitations of the information field need not be exactly equal to the resonance energy of s-atoms in the gain medium. It can deviate from the latter, but not so much. The right formulation of this statement can be done in the probabilistic terms. The emission spectrum of the gain medium has the Gaussian distribution with the mean value $E_{F}$ and sufficiently small standard deviation $\sigma_{A},$ where $\sigma_{A}\frac{T_{2}}{\gamma }<<1.$*

Technically, the condition (38) justifies the following approximation:

(39) $$e^{\pm it\frac{E_{F}-E_{A}}{\gamma }}\approx 1,\;t_{0}\leq t\leq T_{2},$$

in the last two terms of interaction Hamiltonian (25). By taking into account that the first two terms in expression (25) can be neglected due to Assumption 1 interaction Hamiltonian $H_{I},$ can be approximately treated as a time-independent operator.

The condition (38) can be rewritten as

(40) $$|1-\frac{E_{F}}{E_{A}}|<<\frac{\tau_{A}}{T_{2}}.$$

Therefore, for the gain medium which is “good” from the viewpoint of Assumption 1, i.e., $\frac{\tau_{A}}{T_{2}}<<1,$ the lifetime is long and the social energy gap is high, this condition is satisfied only for very sharp Gaussian distribution, with the mean value $E_{F},$ of the emission spectrum of the gain medium.

### 9.5. Probabilistic Consequences of the Quantum Markov Dynamics

Now, by using quantum master Equation (30), we can describe the dynamics of probability p(t,n) to find *n* excitations in social laser’s resonator, e.g., in the form of an Internet Echo Chamber. We have $p\left(t,n\right)=\langle n|\rho_{F}\left(t\right)|n\rangle .$ By averaging Equation (30) with respect to the state |n〉, we obtain the probabilistic dynamics (see [84]):

(41) $$\frac{\partial p}{\partial t}\left(t,n\right)=-A\left[\left(n+1\right)p\left(t,n\right)-np\left(t,n-1\right)\right]-C\left[np\left(t,n\right)-\left(n+1\right)p\left(t,n+1\right)\right]$$

$$+B[{\left(n+1\right)}^{2}p\left(t,n\right)-n^{2}p\left(t,n-1\right)].$$

As in physics, we are interested in the steady state of this dynamics, the state of equilibrium in the Echo Chamber. After approaching this state, the social resonator can be used to emit the powerful wave of social actions. To find a steady state, we set $\frac{\partial p}{\partial t}\left(t,n\right)=0$ and obtain the recurrence equation:

(42) p(n)=(A/C)(1−nB/A)p(n−1).

In spite of simplicity of this recurrence equation, its solution cannot be represented in a compact analytic form. In physics, one considers approximations corresponding different ranges of values of the parameter A/C, *the pump parameter*, representing interrelation of the gain and loss. The equality A/C=1 is *the threshold condition for the laser.*

1. If A/C<1, then the solution of Equation (42) can be approximately represented in the form $p\left(n\right)\approx \left(1-A/C\right){\left(A/C\right)}^{n}.$ Thus, for this region of variation of the parameter A/C, the field is characterized by a small number of excitations: the probability that, in the resonator of the social laser, there can be found *n* excitations decreases exponentially, $p\left(n\right)∼e^{-(\ln C-\ln A)}.$
2. If A/C≈1, then the solution has no simple analytical representation. This range of variation of parameter A/C is characterized by large fluctuations of number *n* of field’s excitations.
3. If A/C>>1, then p(n) can be approximated by the Poission distribution:
  (43) $$p\left(n\right)\approx e^{-\bar{n}}\frac{{\bar{n}}^{n}}{n!},$$
  where $\bar{n}=A^{2}/CB$ is the average number of excitations. It is crucial that the standard deviation of the Poission distribution $\sigma =\sqrt{\bar{n}}.$ This implies that the Gaussian distribution approximating the Poisson distribution with the mean value $\bar{n}=A^{2}/CB>>1$ is concentrated around $\bar{n}.$ Thus (with the high probability), the number of excitations *n* present in the resonator of the social laser is very large.

In some papers, one sets $A/C=e^{b},$ i.e., b=ln(A/C). Then, the threshold value equals to zero.

## 10. Conclusions

This paper is a new step towards clarification of the basic notions of the quantum-like social lasing model. We heavily refer to the information interpretations of quantum mechanics and the operational approach. The latter is based on the Copenhagen interpretation of quantum mechanics and Bohr’s emphasis that quantum theory is about observations and not genuine physical processes in the micro-world. This approach is applied to formalization of the notion of the social (mental) energy. Although this notion has been discussed by psychologists and philosophers [64,65,66] for a few hundred years, its proper formalization was missed. It seems that this notion can be handled properly only in the quantum framework, cf., however, with other recent attempts [69,70,71].

The Bose–Einstein statistics of excitations of the quantum electromagnetic field, photons, plays the crucial role in generation of the cascade process of stimulated emission. Following Schrödinger [75] who used Gibbs ideal ensembles to derive thermodynamical quantities from statistical mechanics, in [47], one of the authors of this paper considered thermodynamics of excitations of the quantum information field. The Bose–Einstein statistics can be derived for them under the assumption of their indistinguishability with respect to the social energy. In this paper, we analyze the meaning of such indistinguishability and emphasize the crucial role of information overload in its creation. The Bose–Einstein statistics of information excitations matches well with one of the basic effect of social psychology, the bandwagon effect. We also present briefly thermodynamical derivation of the Bose–Einstein statistics for information excitations [47] by emphasizing the operational meaning of the notion of the social energy.

We discuss in a lot of detail the notion of the social color of an excitation of the quantum information field. This quantity represents characteristics of information excitations linked to social lasing coherence. The role of the social color in the process of energy pumping versus amplified coherent emission is clarified.

As in physical lasing, a resonator is the basic component of social lasing. The standard resonator of a physical laser is the optical cavity. Therefore, modeling of functioning of a physical laser resonator is typically presented in the framework of quantum optics. Our aim was to “distill” the physical scheme from connection with optics. We proceeded with a theory of open quantum systems and the quantum Markov approximation (given by the GKSL-equation) of the dynamics of the compound system, *s*-atoms interacting with the quantum information field. As is typical in quantum-like modeling, we borrowed the mathematical formalism of quantum physics, but assigned new (social) interpretations to the basic quantities and parameters. This interpretational analysis of the (standard) mathematical expressions highlights the following social lasing constraints on the human gain medium and the quantum information field:

- A gain medium is characterized by the long lifetime of the excited state and the big gap between the states of relaxation and excitation.
- The social energy carried by excitations of the information field has to match the resonance energy of *s*-atoms in the gain medium.
- The interrelation of the magnitudes of the excitation and absorption rates $r_{2},r_{1}$ and the lifetimes of the corresponding levels $T_{2},T_{1}$ has to imply inequality A>C, where *A* and *C* are gain and loss coefficients, respectively.
- The nonlinear character of interactions between excitations in a social laser resonator (encoded in the *B*-coefficient) plays the crucial role in initiation of stable social lasing.
- The quantum-like regime of lasing is characterized by the threshold value of the pump parameter.

The presented study on the foundational side of the quantum-like modeling of social lasing is important for its further development, since, as was mentioned in the Introduction, application of the quantum theory outside physics requests reanalyzing of quantum methodology.

This paper may even have some impact for quantum foundations, especially the information interpretations of quantum mechanics [51,52,53,54,55,56,57,58,59,60,61,62,63]. The application of the mathematical formalism of quantum mechanics outside physics supports (at least indirectly) the claim that this formalism basically reflects the special laws of information processing. (The latter is confirmed by its derivation from the natural informational principles [57,58,59].) In particular, the thermodynamical derivation of quantum statistics (Section 4.4) highlights the role of indistinguishability of information quanta.
